# Keeping time on liver fibrosis

**DOI:** 10.1038/s41401-021-00716-2

**Published:** 2021-08-11

**Authors:** Steven O’Reilly

**Affiliations:** https://www.nature.com/ActaPharmacolSin

Liver fibrosis is a common and deadly consequence of repeated exposure to an insult which can be of varying aetiology including viral (hepatitis), autoimmune or alcohol misuse. Key to the disease is the activation of stellate cells that produce a surfeit of extracellular matrix (ECM) including collagens and fibronectin and express the contractile protein alpha-smooth muscle actin. It is now accepted that to activate these key ECM-producing cells new substrates and energy are needed, through glycolysis this is performed. Recently, Xu et al demonstrated a link between phenotypic conversion, glycolysis and the circadian clock [1].

The circadian clock is a cell intrinsic clock that is a timekeeper. Circadian clocks in the brain and peripheral tissues temporally coordinate local context to align with the 24-h  rhythmic environment through light/darkness cycles [2]. It is known in shift workers that they have a higher incidence of disease, suggesting that disruptions of the circadian clock mediate disease. Indeed immune cells such as monocytes and macrophages display a robust molecular clock and at least 8% of transcripts in murine macrophages are circadian [3]. Indeed the time of day that the mice are challenged will give a divergent response [4]. One key component of the molecular clock is the transcription factor Brain and Muscle ARNT-Like 1 (BMAL1). BMAL1 is a transcription factor that binds with CLOCK to positively regulate circadian genes and regulate biological functions. This heterodimers also activate their transcriptional repressors Period1 and Period2 and Cryptochrome 1 and Crytopchrome 2 (Cry1/2) via a negative feedback loop

In Xu’s study they demonstrated significantly reduced BMAL1 levels in the classic carbon tetrachloride model of liver fibrosis in mice–a standard model for in vivo liver fibrosis. They also showed significant reduction in BMAL1 in vitro in hepatic stellate cells and in LX-2 cells that the classic fibrotic cytokine TGF-β1 reduced BMAL1 expression at the protein level suggesting TGF-β1 is an upstream regulator of BMAL1. Using an overexpression vector to artificially enhance BMAL1 they demonstrated downregulation of key glycolysis enzymes Hexokinase II (HKII) and PKM2 and a subsequent reduction in the glycolytic rate and finally they demonstrated overexpression of BMAL1 retarded the phenotypic conversion of the stellate cells and ECM production.

Furthermore, they found that BMAL1 interacts with the protein isocitrate dehydrogenase-1 (IDH-1). IDH-1 is the key enzyme in the decarboxylation of isocitrate to yield α-ketoglutarate in the TCA cycle. IDH-1 was also reduced by TGF-β1 treatment in vitro. Overexpression of BMAL1 resulted in a large increase in IDH-1 and a significant increase in its product α-ketoglutarate and a concomitant reduction in glycolysis (and HKII) which could be reversed with small interfering RNA to IDH-1. Finally using a recombinant adenoviral associated viral system they overexpressed BMAL1 specifically in hepatocytes and used the carbon tetrachloride model of fibrosis. This hepatocyte overexpression of BMAL1 significantly reduced upregulated glycolysis, reduced key glycolytic enzymes and elevated IDH-1. Most importantly this retarded liver fibrosis and collagen deposition are associated with increased α-ketoglutarate. Because BMAL1 inhibitory effect on glycolysis was abolished by IDH-1 and therefore α-ketoglutarate silencing, this demonstrate that α-ketoglutarate is required for BMAL1’s antifibrotic effect.

How is α-ketogluturate retarding glycolysis? This molecule normally serves as a precursor for amino acid synthesis and as a substrate for 2-oxygluturate-dependant dioxygenases which include epigenetic enzymes that belong to the histone demethylase family. These histone demethylases remove methyl marks on histones and thereby regulate gene expression. It could therefore be speculated that α-ketogluturate increased expression/activity of histone demethylase enzymes that results in repression of glycolysis. In respect of a recent study, albeit in endothelial cells, it was demonstrated that the Jumanji-domain-containing protein 8 protein, which used α-ketoglutarate as a substrate, regulated glycolysis by direct regulation of the key glycolytic enzyme PKM2 [5].

This work demonstrates that the circadian clock protein BMAL1 is an antifibrotic molecule via regulation of glycolysis through the IDH-1/α-ketoglutarate axis. Strategies that restore reduced BMAL1 could be a therapeutic approach in liver fibrosis–a disease with high unmet medical need.
